# Supplementary material for: Defined factors to reactivate cell cycle activity in adult mouse cardiomyocytes
Source: Sci Rep. 2019 Dec 11;9:18830. doi: 10.1038/s41598-019-55027-8 (PMC6906479; doi:10.1038/s41598-019-55027-8)
Supplement: Supplementary file 1 — Supplemental figures S1-9 and tables S2-4 [file 41598_2019_55027_MOESM1_ESM.pdf]

Supplemental Material

Defined factors to reactivate cell cycle activity in adult mouse cardiomyocytes

[1]Justin Judd, [1]Jonathan Lovas, [1, 2, 3 #]Guo N. Huang

[1]Cardiovascular Research Institute, [2]Department of Physiology, [3] Eli and Edythe Broad Center of Regeneration Medicine and Stem Cell Research, University of California, San Francisco, San Francisco CA 94158, USA

# Corresponding author: Cardiovascular Research Institute, School of Medicine, University of California, San Francisco. 555 Mission Bay Boulevard South, San Francisco, CA 94158 Tel: 415-502-2879. Email: Guo.Huang@ucsf.edu

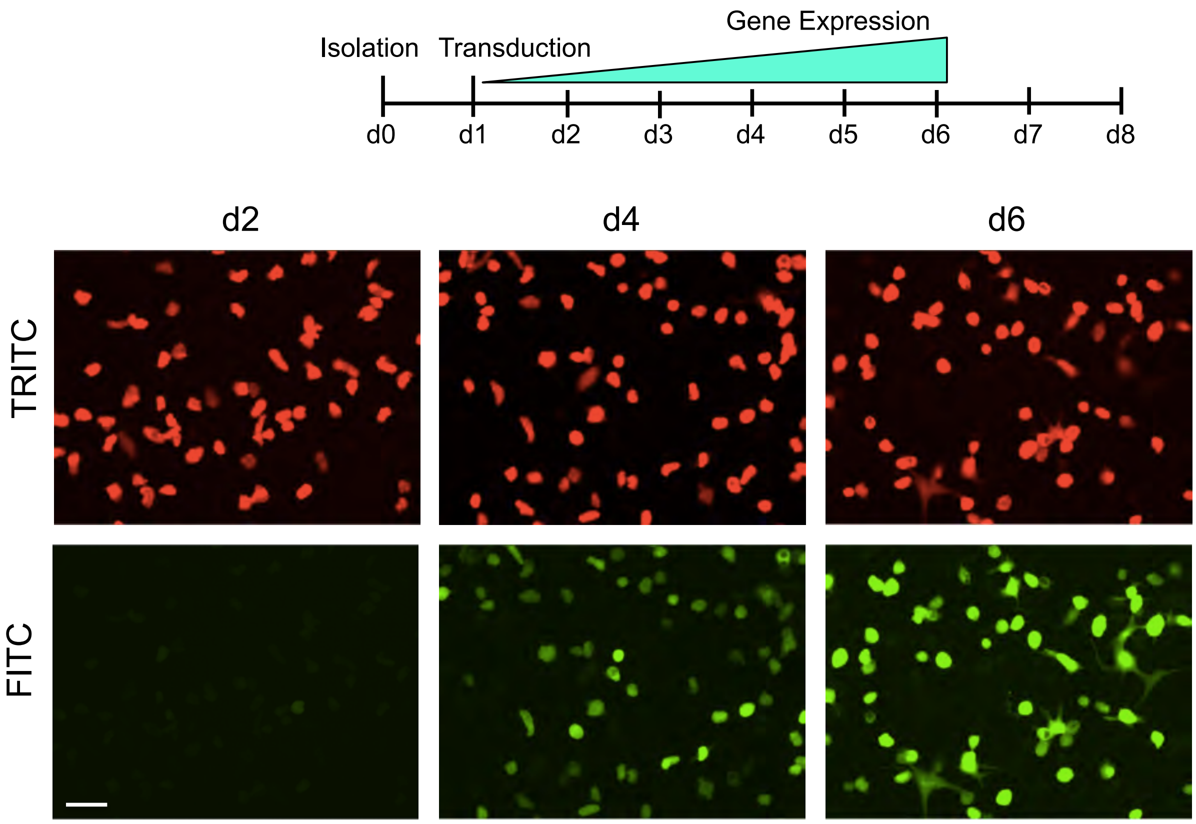

Figure S1. Transduction of cardiomyocytes by adenovirus expressing GFP. Lineage traced tdTomato^{+} adult mouse cardiomyocytes are transduced with Ad-GFP at day 1 post-isolation. Gene expression is detectable, but very low at d2 (d1 post-transduction), but robust at d4 and very strong by d6. Scale bars, 100 µm.

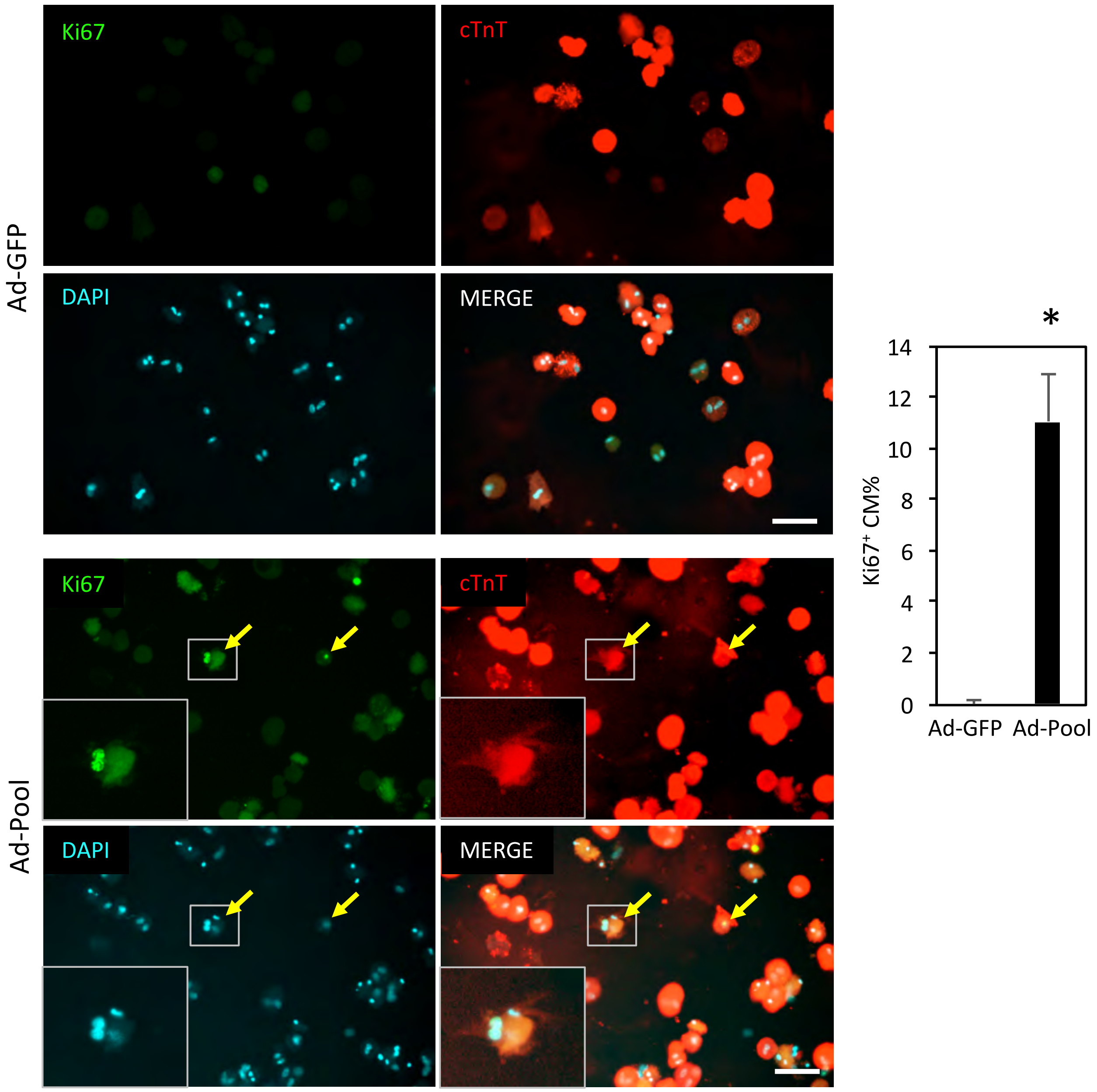

Figure S2. Increase of cardiomyocyte proliferation by pooled adenoviruses expressing all candidate genes. Adult mouse cardiomyocytes are transduced with viruses at day 1 post-isolation and stained at day 4. Yellow arrows point at Ki67-positive cardiomyocytes. High-magnification views of the boxed areas are shown in the insets. *, *p*<0.05. Scale bars, 100 µm.

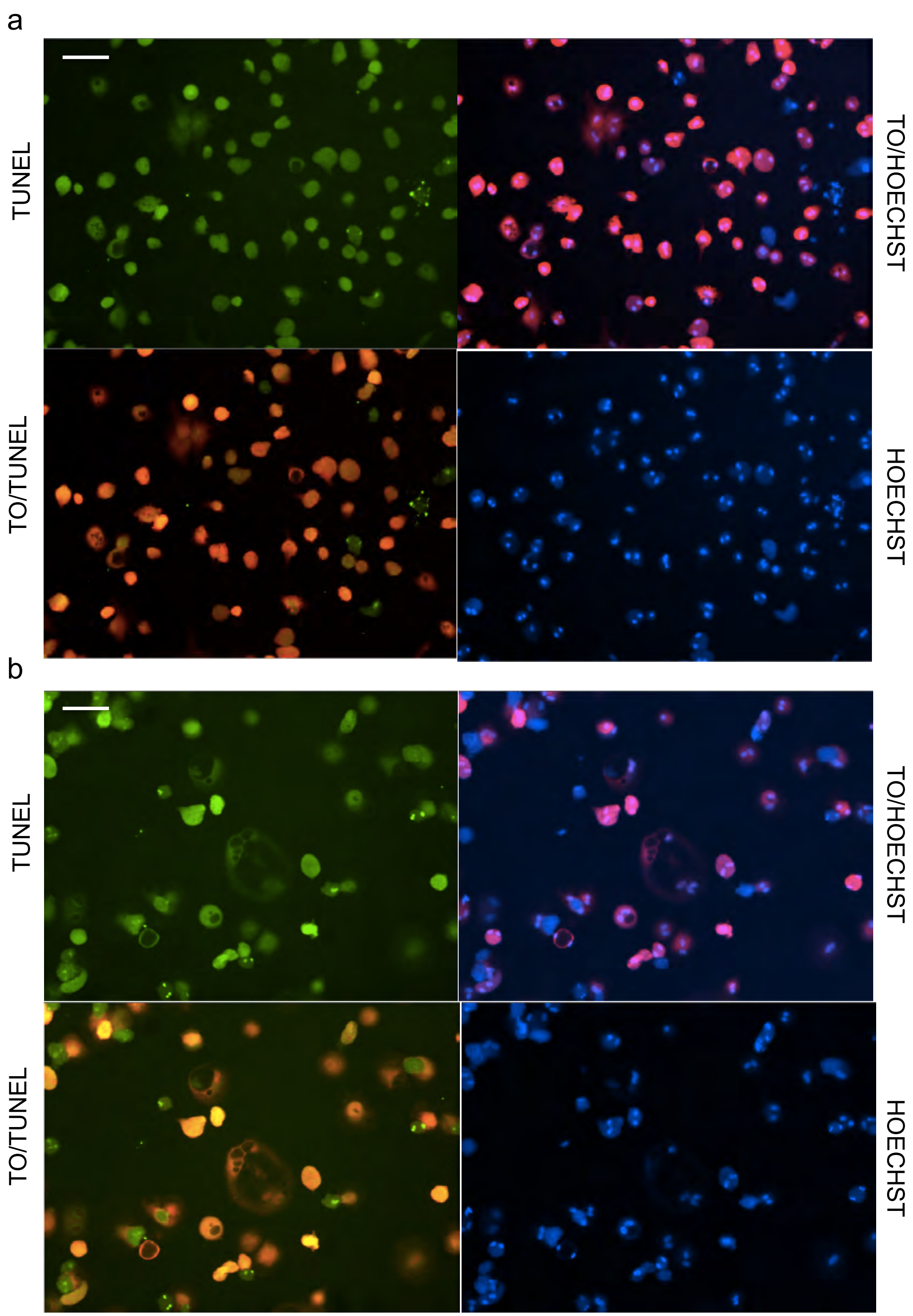

Figure S3. TUNEL staining of control and E2F2-treated cardiomyocytes. (a) TUNEL staining of untreated negative control cardiomyocytes shows low incidence of genomic degradation. (b) E2F2-treated cardiomyocytes by contrast show significant TUNEL label. Scale bars, 100 µm.

	Pool/SubPool (✔ = included in subpool)				
	1	1.1	1.2	1.3	1.4
WFDC2	✔	✔	✔	✔	
UBE2L6	✔	✔	✔	✔	
GYG1	✔	✔	✔	✔	
PRC1	✔	✔	✔	✔	
FOXM1	✔	✔	✔		✔
FAM60A	✔	✔	✔		✔
MDK	✔	✔	✔		✔
RPS27A	✔	✔	✔		✔
Tceal8	✔	✔		✔	✔
GOLM1	✔	✔		✔	✔
Bmp4	✔	✔		✔	✔
TUBB2B	✔	✔		✔	✔
ELOF1	✔		✔	✔	✔
E2f2	✔		✔	✔	✔
PLK1	✔		✔	✔	✔
ERH	✔		✔	✔	✔
TNFRSF12A	✔		✔	✔	✔

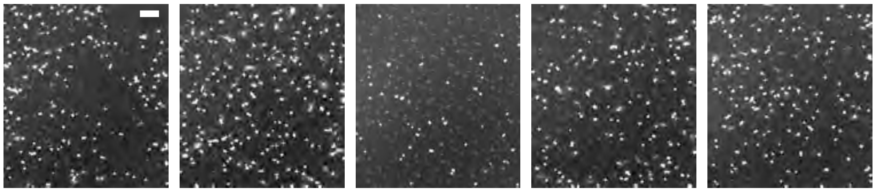

Subpool 1 | Subpool 1.1 | Subpool 1.2 | Subpool 1.3 | Subpool 1.4

Figure S4. Identification of candidate genes that rescue viability from E2F2-mediated cell death. Each pool contains 12-17 adenoviruses overexpressing indicated genes (indicated with check symbols). Genes removed from each SubPool (1.1, 1.2, etc.) of Pool 1 are indicated by grey boxes. Only gene subpool1.2 was insufficient to rescue viability in the presence of E2F2 overexpression. Scale bars, 200 µm.

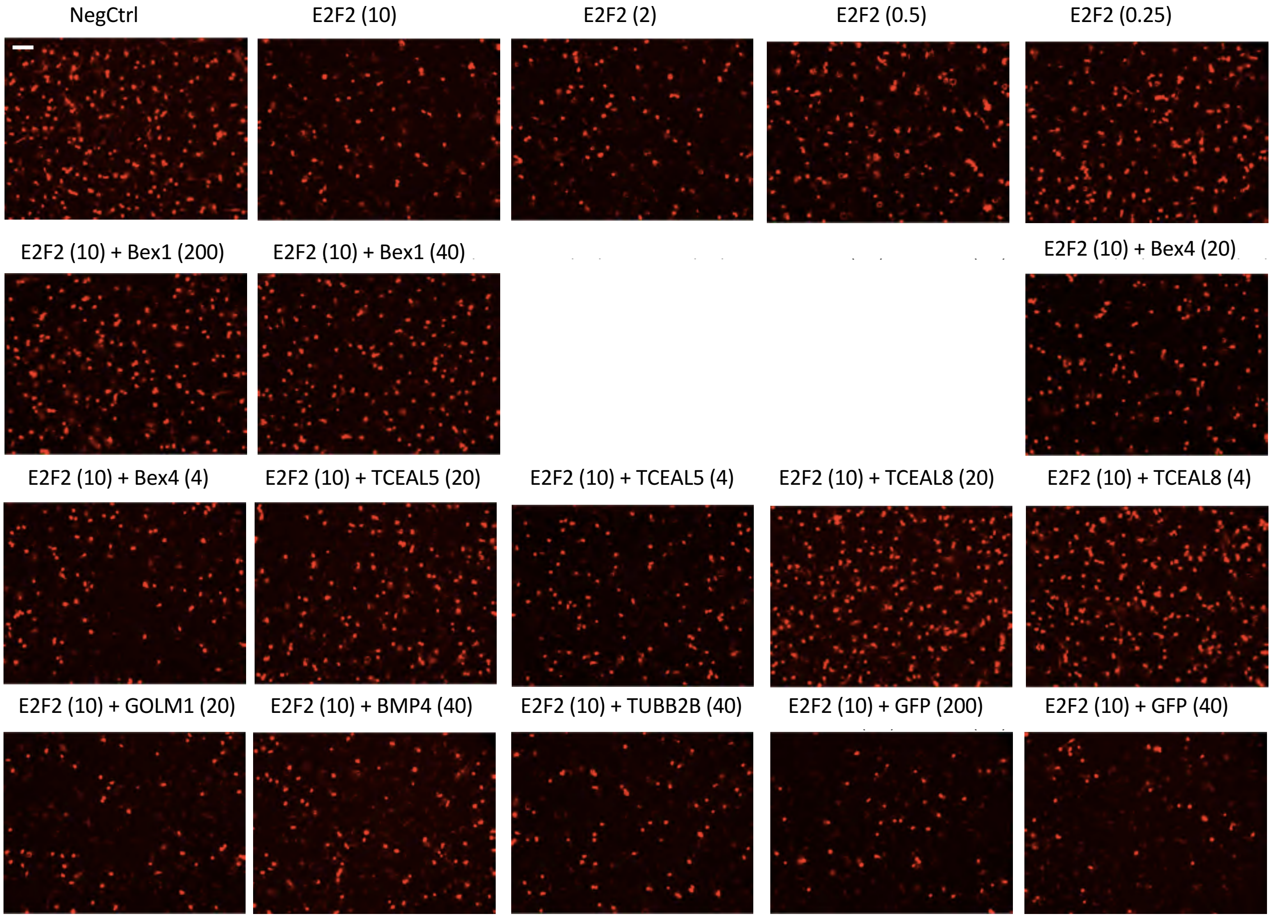

Figure S5. Screening BEX superfamily genes for inhibition of E2F2-mediated cell death. (Top row) E2F2 was expressed by adenovirus vector at various MOI. Cell viability is visualized by the transgenic tdTomato expression (red) at day 7 post-transduction. BEX superfamily genes and other candidate genes were co-expressed with E2F2. Note only TCEAL8 and BEX1 exhibit strong inhibition of cell death. MOI is indicated in parentheses. Scale bars, 200 μm.

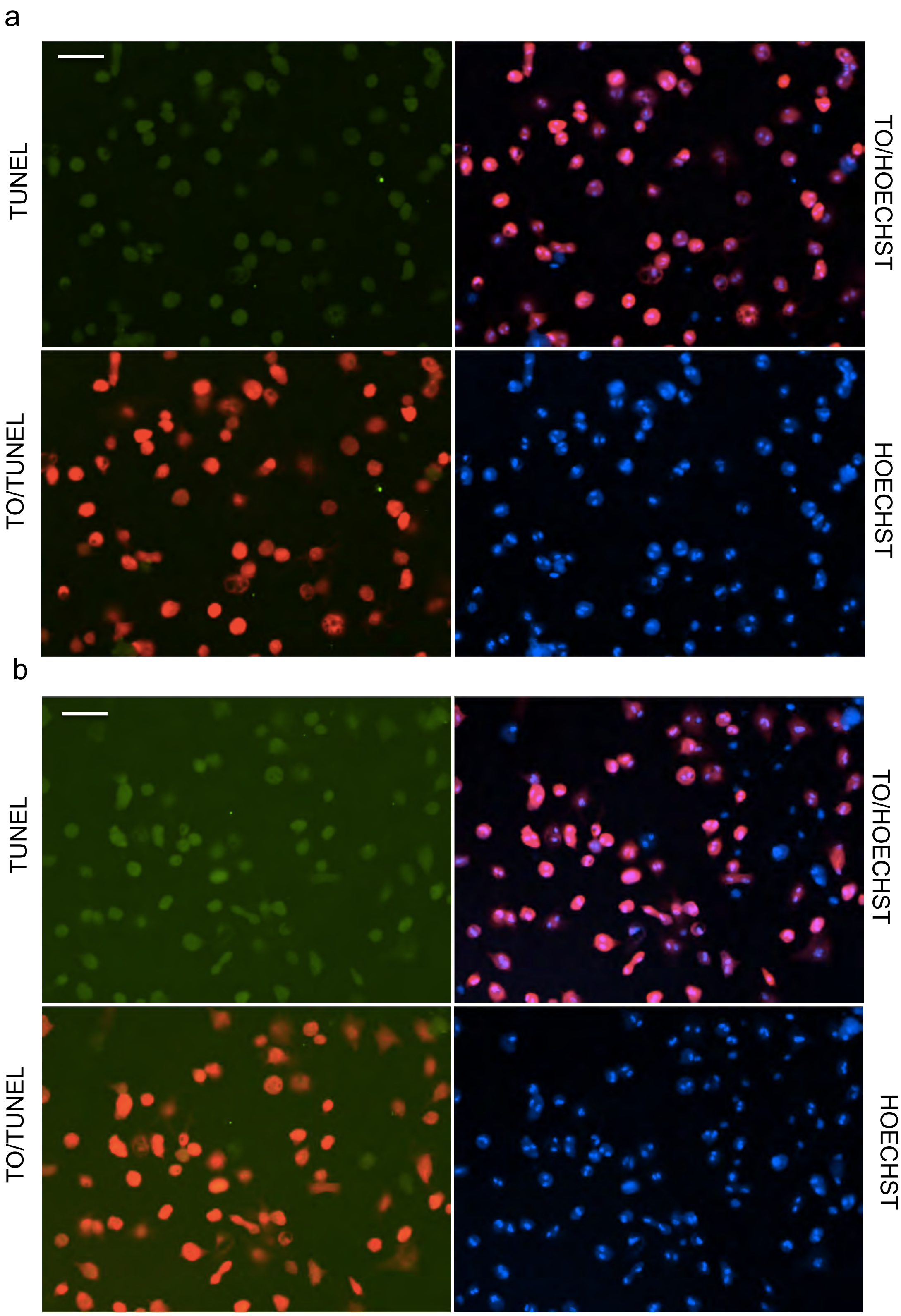

Figure S6. TUNEL staining of E2F2-treated cardiomyocytes co-expressed with E2F2 and TCEAL8 or BEX1. TUNEL labeling shows little genomic degradation at day 5 post-transduction in E2F2-treated cardiomyocytes when co-expressed with TCEAL8 (a) or BEX1 (b). Scale bars, 100 µm.

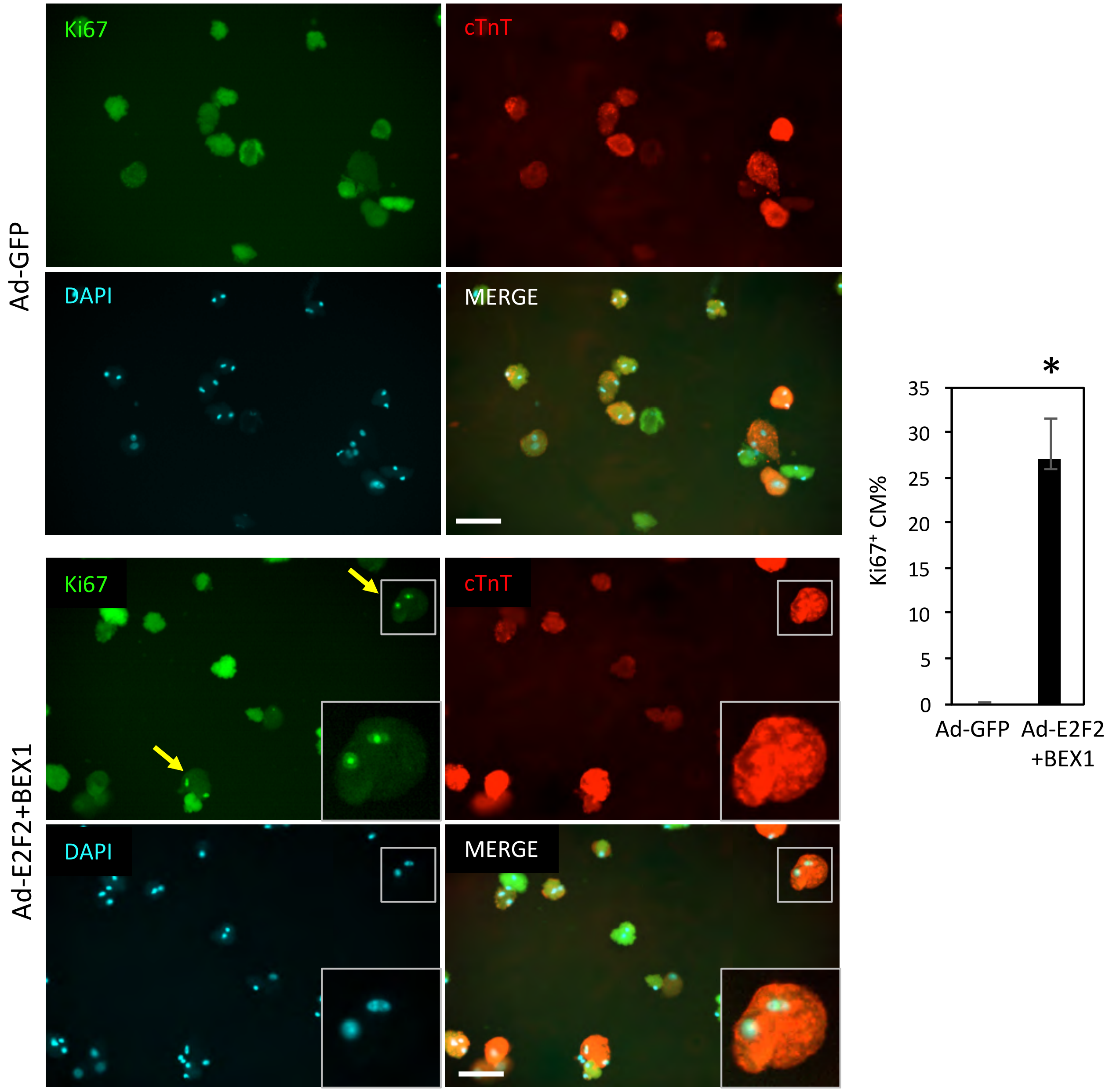

Figure S7. Increase of cardiomyocyte proliferation by adenoviruses E2F2 and BEX1. Adult mouse cardiomyocytes are transduced with viruses at day 1 post-isolation and stained at day 4. Yellow arrows point at Ki67-positive cardiomyocytes. High-magnification views of the boxed areas are shown in the insets. *, p<0.05. Scale bars, 100 µm.

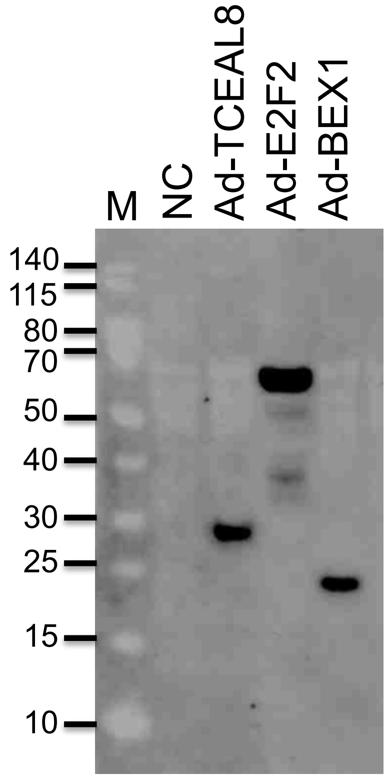

Figure S8. Western blot analysis of V5-tagged E2F2, BEX1, and TCEAL8 protein expression in HEK 293T cells. (a) Expression of TCEAL8-V5, E2F2-V5, and BEX1-V5 proteins were confirmed by western blot analysis of HEK 293T cell lysate at 1 day post-infection, at relative MOIs 1, 2.5, 40, respectively (normalized to Ad-Tceal8). NC: negative control, no virus. Blot was stained with an anti-V5 antibody. Scale bars, 100 µm.

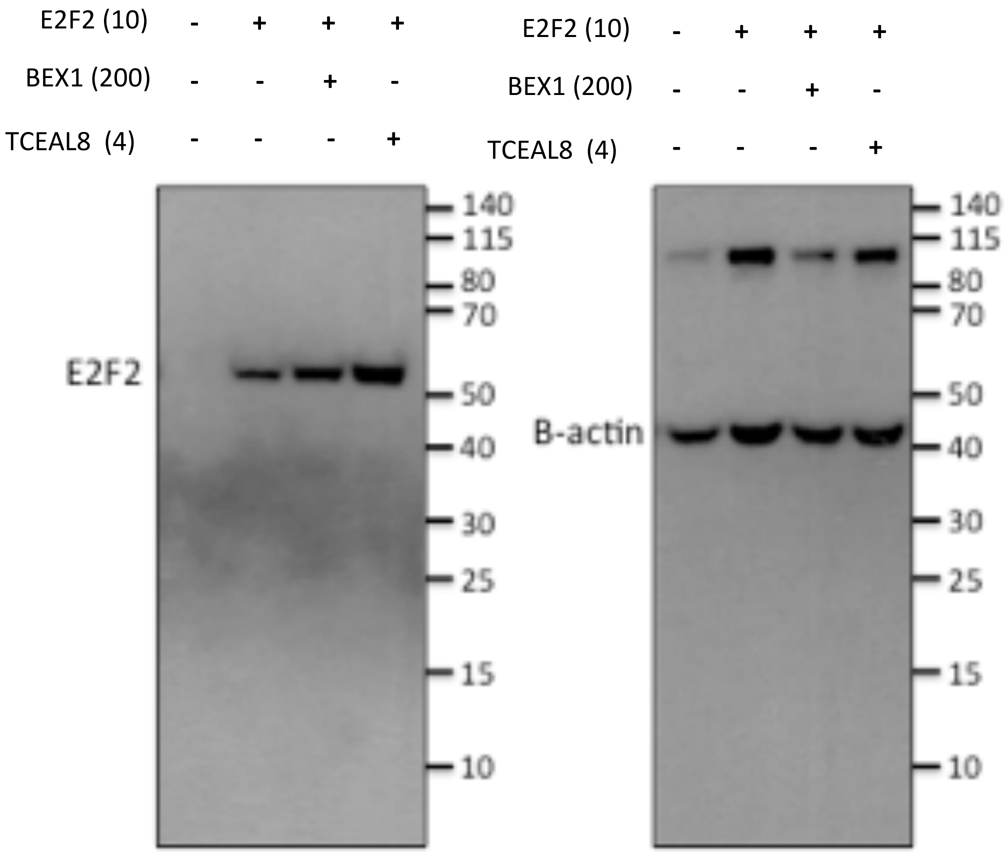

Figure S9. Western blot analysis showing E2F2 co-expressed with Tceal8 and Bex1. Full blot images corresponding to Figure 4b.

Table S2. Genes selected for functional tests

Genes picked because they are selectively more downregulated in the adult myocardium than that in the adult epicardium (low values in the B column).

B: Heart/Epi(Dev Index)=C/D

C: Heart (Adult/E11.5)=E/G

D: Epi (Adult/E11.5)=F/H

	A	B	C	D	E	F	G	H
#	**Genes**	**Heart/Epi (Dev Index)**	**Heart (Adult/E11.5)**	**Epi (Adult/E11.5)**	**Adult Total Heart**	**Adult Epi**	**E11.5 heart**	**E11.5 epi**
1	Bex4	0.04	0.02	0.56	138	897	6068	1593
2	Tuft1	0.04	0.04	0.93	215	1107	5719	1192
3	Ube2l6	0.05	0.06	1.33	167	1020	2625	768
4	Wfdc2	0.05	0.13	2.60	75	1741	574	671
5	Gpc3	0.05	0.04	0.65	282	2714	7970	4151
6	E2f2	0.08	0.03	0.43	75	197	2153	454
7	Hmgb2	0.09	0.07	0.78	248	334	3624	427
8	Ptn	0.09	0.02	0.27	163	1089	6696	4087
9	Anln	0.11	0.06	0.57	98	241	1538	425
10	Slc43a1	0.12	0.04	0.35	95	341	2330	975
11	Fgfr2	0.12	0.08	0.64	132	468	1669	728
12	Capn6	0.13	0.01	0.11	82	581	5652	5178
13	Golm1	0.14	0.27	2.01	413	2271	1515	1132
14	Cited4	0.15	0.09	0.62	704	660	7816	1067
15	Bmp4	0.18	0.17	0.94	260	824	1544	873
16	Smarce1	0.19	0.22	1.15	413	815	1850	709
17	Prc1	0.19	0.02	0.10	82	104	4023	994
18	Cenpw	0.23	0.05	0.23	107	348	2035	1547
19	H2afz	0.26	0.02	0.06	140	416	8885	6816
20	Tubb2b	0.28	0.03	0.11	262	794	8796	7378
21	Itm2a	0.30	0.10	0.32	465	2244	4865	7011
22	Rps27a	0.31	0.27	0.87	893	2737	3305	3136
23	Ngfrap1	0.31	0.06	0.19	464	1404	7999	7572
24	Pitx2	0.32	0.13	0.40	115	155	889	387
25	Ezh2	0.32	0.16	0.50	216	203	1339	409
26	Ndn	0.34	0.12	0.34	818	2907	7095	8577
27	Mdk	0.45	0.02	0.04	329	1045	18938	26948
28	Bmp7	0.45	0.13	0.29	55	84	419	295
29	Stxbp2	0.49	0.09	0.18	165	573	1914	3258
30	Efs	0.50	0.26	0.51	162	569	636	1107
31	Gyg1	0.53	0.36	0.68	903	943	2487	1383
32	Tmsb10	0.54	0.08	0.14	462	1772	5913	12243
33	Smarcc1	0.54	0.19	0.36	145	111	745	311
34	Sec61b	0.55	0.34	0.62	341	1096	1003	1767
35	Erh	0.59	0.17	0.28	771	1222	4665	4391
36	Mfap2	0.70	0.04	0.06	70	222	1677	3735
37	Elof1	0.73	0.34	0.46	423	1351	1262	2929
38	Tuba1a	0.77	0.10	0.14	1049	2084	10026	15333

Genes selected by literature search.

39	Igf2	0.96	0.18	0.19	4247	3748	23705	20175
40	E2f1	1.17	0.09	0.08	59	86	651	1117
41	Foxm1	1.20	0.17	0.14	60	68	353	485
42	Plk1	1.26	0.05	0.04	68	77	1488	2113
43	Sox12	1.51	0.19	0.13	481	645	2519	5103
44	Tnfrsf12a	1.75	1.26	0.72	514	806	407	1114
45	Tead2	2.01	0.06	0.03	214	201	3464	6535

Genes not present in our microarray but selected by literature search.

46	Tns3
47	Lin28b
48	Fam60a

Additional Bex superfamily genes selected for analysis (not present in our microarray).

49	Bex1
50	Tceal8
51	Wbp5 (Tceal9)
52	Tceal5

Table S3. Final gene list

Gene 1-17: Pool 1; Gene 18-36: Pool 2 (see Figure 2c).

Gene #	Gene Symbol	Species	Accession ID	Pool #	Source of Candidate Gene Selection
1	WFDC2	H. sapiens	NM_006103.3	Gene 1-17	Microarray
2	UBE2L6	H. sapiens	NM_004223.4	Gene 1-17	Microarray
3	GYG1	H. sapiens	NM_004130.3	Gene 1-17	Microarray
4	PRC1	H. sapiens	NM_003981.3	Gene 1-17	Microarray
5	FOXM1	H. sapiens	NM_202002.2	Gene 1-17	Microarray
6	FAM60A	H. sapiens	NM_001135811.1	Gene 1-17	Literature Search
7	MDK	H. sapiens	NM_001012334.2	Gene 1-17	Microarray
8	RPS27A	H. sapiens	NM_002954.5	Gene 1-17	Microarray
9	Tceal8	M. Musculus	NM_001168578.1	Gene 1-17	Homolog of Bex4
10	GOLM1	H. sapiens	NM_016548.3	Gene 1-17	Microarray
11	Bmp4	M. Musculus	NM_007554.3	Gene 1-17	Microarray
12	TUBB2B	H. sapiens	NM_178012.4	Gene 1-17	Microarray
13	ELOF1	H. sapiens	NM_032377.3	Gene 1-17	Microarray
14	E2f2	M. Musculus	NM_177733.7	Gene 1-17	Microarray
15	PLK1	H. sapiens	NM_005030.5	Gene 1-17	Microarray
16	ERH	H. sapiens	NM_004450.2	Gene 1-17	Microarray
17	TNFRSF12A	H. sapiens	NM_016639.2	Gene 1-17	Microarray
18	MFAP2	H. sapiens	NM_017459.2	Gene 18-36	Microarray
19	PITX2	H. sapiens	NM_000325.5	Gene 18-36	Microarray
20	TUFT1	H. sapiens	NM_001126337.1	Gene 18-36	Microarray
21	STXBP2	H. sapiens	NM_006949.3	Gene 18-36	Microarray
22	CAPN6	H. sapiens	NM_014289.3	Gene 18-36	Microarray
23	ITM2A	H. sapiens	NM_004867.4	Gene 18-36	Microarray
24	SFDC2	H. Sapiens	NM_006103.3	Gene 18-36	Microarray
25	TCEAL9	H. sapiens	NM_016303.2	Gene 18-36	Homolog of Bex4
26	NDN	H. sapiens	NM_002487.2	Gene 18-36	Microarray
27	TNS3	H. sapiens	NM_022748.11	Gene 18-36	Literature Search
28	BEX1	H. sapiens	NM_018476.3	Gene 18-36	Homolog of Bex4
29	SEC61B	H. sapiens	NM_006808.2	Gene 18-36	Microarray
30	FGFR2	H. sapiens	NM_000141.4	Gene 18-36	Microarray
31	ANLN	H. Sapiens	NM_018685.4	Gene 18-36	Microarray
32	EFS	H. sapiens	NM_032459.2	Gene 18-36	Microarray
33	TCEAL5	H. sapiens	NM_001012979.3	Gene 18-36	Homolog of Bex4
34	CITED4	H. sapiens	NM_133467.2	Gene 18-36	Microarray
35	LIN28B	H. sapiens	NM_001004317.3	Gene 18-36	Literature Search
36	PTN	H. Sapiens	NM_002825.5	Gene 18-36	Microarray

Table S4. Primer list

**Primer name**	**Primer sequence**
p19-ARF_F	gtcgcaggttcttggtcact
p19-ARF_R	cgaatctgcaccgtagttga
CyclinA2_F	cttggctgcaccaacagtaa
CyclinA2_R	atgactcaggccagctctgt
CyclinD2_F	ttacctggaccgtttcttgg
CyclinD2_R	tgctcaatgaagtcgtgagg
CyclinE_F	cctccaaagttgcaccagtt
CyclinE_R	ggacgcacaggtctagaagc
mdm2_F	tgcaagcacctcacagattc
mdm2_R	acacaatgtgctgctgcttc
p21CIP/WAF_F	cggtggaactttgacttcgt
p21CIP/WAF_R	cagggcagaggaagtactgg
GAPDH_F	gcagtggcaaagtggagattg
GAPDH_R	agagatgatgacccttttggctcc
